# Phylogeography of *Camellia taliensis* (Theaceae) inferred from chloroplast and nuclear DNA: insights into evolutionary history and conservation

**DOI:** 10.1186/1471-2148-12-92

**Published:** 2012-06-21

**Authors:** Yang Liu, Shi-xiong Yang, Peng-zhang Ji, Li-zhi Gao

**Affiliations:** 1 Plant Germplasm and Genomics Center, Germplasm Bank of Wild Species, Kunming Institute of Botany, Chinese Academy of Sciences, Kunming, 650204, China; 2 Key Laboratory of Biodiversity and Biogeography, Kunming Institute of Botany, Chinese Academy of Sciences, Kunming, 650204, China; 3 Tea Research Institute, Yunnan Academy of Agricultural Sciences, Menghai, 666201, P. R. China

## Abstract

**Background:**

As one of the most important but seriously endangered wild relatives of the cultivated tea, *Camellia taliensis* harbors valuable gene resources for tea tree improvement in the future. The knowledge of genetic variation and population structure may provide insights into evolutionary history and germplasm conservation of the species.

**Results:**

Here, we sampled 21 natural populations from the species' range in China and performed the phylogeography of *C. taliensis* by using the nuclear *PAL* gene fragment and chloroplast *rpl32-trnL* intergenic spacer. Levels of haplotype diversity and nucleotide diversity detected at *rpl32-trnL* (*h* = 0.841; *π* = 0.00314) were almost as high as at *PAL* (*h* = 0.836; *π* = 0.00417). Significant chloroplast DNA population subdivision was detected (*G*_ST_ = 0.988; *N*_ST_ = 0.989), suggesting fairly high genetic differentiation and low levels of recurrent gene flow through seeds among populations. Nested clade phylogeographic analysis of chlorotypes suggests that population genetic structure in *C. taliensis* has been affected by habitat fragmentation in the past. However, the detection of a moderate nrDNA population subdivision (*G*_ST_ = 0.222; *N*_ST_ = 0.301) provided the evidence of efficient pollen-mediated gene flow among populations and significant phylogeographical structure (*N*_ST_ > *G*_ST_; *P* < 0.01). The analysis of *PAL* haplotypes indicates that phylogeographical pattern of nrDNA haplotypes might be caused by restricted gene flow with isolation by distance, which was also supported by Mantel’s test of nrDNA haplotypes (*r* = 0.234, *P* < 0.001). We found that chlorotype C1 was fixed in seven populations of Lancang River Region, implying that the Lancang River might have provided a corridor for the long-distance dispersal of the species.

**Conclusions:**

We found that *C. taliensis* showed fairly high genetic differentiation resulting from restricted gene flow and habitat fragmentation. This phylogeographical study gives us deep insights into population structure of the species and conservation strategies for germplasm sampling and developing *in situ* conservation of natural populations.

## Background

Crop wild relatives, which include wild progenitors of cultivated plants as well as other closely related species, are important components of natural habitats and agroecosystem [1]. They may contain desirable alleles that can enhance pest/disease resistance and abiotic adaptation, or improve nutritional values or flavour of crops, providing plant breeders with a broad pool of potentially useful genetic resources. Wild relatives have repeatedly served as important sources of useful traits for genetically impoverished crops in the past decades [2]. However, these wild species are subject to an increasing range of threats in their natural habitats [3]. To design optimal conservation and management strategies, it is necessary to trace their evolutionary histories by better investigating their population structure.

Phylogeographical studies have been emerging as a powerful tool for understanding population structure and evolution of plant species [4,5]. By synthesizing the influence of both history and current genetic exchanges, phylogeography uses genealogical and geographical information to infer the demographic and historical processes that shaped the evolution of populations and species [5,6]. Recent decades have witnessed increasing applications of phylogeography in economically important plants and their wild relatives [2,7-12].

The genus *Camellia* is composed of over 110 taxa [13], of which only one species, *C. sinensis* (L.) O. Kuntze, is commercially used as a source of the beverage tea. *C. taliensis* is one of the most important wild relatives of the cultivated tea, and they together belong to the section *Thea*. Both them are monoecious, insect-pollinated, and outcrossing species. They differ primarily in the number of locules and the size of flowers and leaves. The number of locules per ovary is five in *C. talensis*, while *C. sinensis* has three. *C. taliensis* usually grows in the mountainous evergreen broad-leaved forests at altitudes from 1300 to 2700 m, and is mainly distributed in southwestern Yunnan of China, as well as adjacent regions including northern Myanmar and Thailand. Because of its close relationship with the cultivated tea and fascinated aftertaste, *C. taliensis* also has been consumed instead of regular tea by local people in parts of Asia, particularly in Yunnan Province of China [13]. These wild tea germplasms undoubtedly harbor abundant gene sources and thus possess great potentials to enhance genetic improvement of cultivated tea in the future. Unfortunately, recent human overexploitation to subtropical forests has unavoidably made *C. taliensis* suffer the degradation and fragmentation of natural habitats suitable for natural populations of the species. In particular, the well-known Pu’er tea, made from organic leaves of wild *C. taliensis* plants in Yunnan Province, enjoys a price 10–100 times higher than cultivated tea trees in the market. For this reason, natural populations of the species have become endangered due to over-picking driven by economic incentives. Hence, it is urgently needed to pay particular attention to making efficient germplasm conservation of *C. taliensis*.

The use of molecular markers derived from nuclear and chloroplast genomes provides an unprecedented opportunity to investigate genetic structure and take insights into population history of a species, particularly by means of a combined analysis of biparentally inherited nuclear and maternally inherited organelle markers [4,5,14-16]. In order to better elucidate population genetic structure and the phylogeography of *C. taliensis*, nucleotide polymorphisms of a total of 21 natural populations (Table 1) were screened by using the maternally inherited cpDNA *rpl32-trnL* spacer and biparentally inherited nuclear *PAL* gene fragment. It was shown that *rpl32-trnL* intergenic spacer could offer higher level of variation than other 33 noncoding regions in the chloroplast, and thus has been successfully applied in phylogeography analyses of many plants [17-19]. Phenylalanine ammonialyase (*PAL*) was reported to involve in the phenylpropanoid pathway and is encoded by a single-copy gene [20]. As a result, it has often been used to evaluate genetic diversity of cultivated tea and elucidate the differentiation among cultivars [21-23]. In this study, we aim to: (i) investigate levels of genetic diversity and genetic structure of *C. taliensis* populations; (ii) infer evolutionary forces that might have shaped the observed population structure and determine demographic and evolutionary history; and (iii) propose efficient strategies for guiding the future germplasm preservation actions and *in situ* conservation management.

**Table 1:** **The sample sizes and geographical locations of the *C. taliensis *populations in this study**

**Population code**	**Geographical origins**	**Latitude (N)**	**Longitude (E)**	**Sample Sizes**	
				***rpl32- trnL***	**PAL**
**1. BD**	Bada, Menghai	21°50´	100°06´	8	5
**2. CNB**	Bajiaozhai, Changning	24°45´	99°47´	9	7
**3. CNC**	Chashanhe, Changning	24°58´	99°36´	9	9
**4. CY**	Shanjia, Cangyuan	23°11´	99°22´	10	7
**5. FQ**	Xiangzhuqing, Fengqing	24°36´	100°09´	9	7
**6. GM**	Manghong, Gengma	23°33´	99°35´	8	5
**7. JC**	Yaoren Mountain, Jiangchen	22°30´	102°01´	7	8
**8. MD**	Lexin, Kachin State, Myanmar	24°49´	97°44´	12	4
**9. MJ**	Yayi, Mojiang	23°11´	101°43´	9	7
**10. NE**	Baicaodi, Ninger	23°15´	100°04´	7	8
**11. SJ**	Mengku, Shuangjiang	23°41´	99°47´	7	6
**12. TCB**	Gaoligong Mountain, Tengchong	24°55´	98°45´	10	6
**13. TCD**	Gaoligong Mountain, Tengchong	24°56´	98°44´	10	7
**14. TCH**	Houqiao, Tengchong	25°17´	98°07´	8	6
**15. XM**	Mengka, Ximeng	22°44´	99°26´	10	5
**16. YD**	Tanglishan, Yongde	24°02´	99°13´	9	7
**17. YJ**	Yangchajie, Yuanjiang	23°40´	101°45´	7	6
**18. YJM**	Mengnong, Yingjiang	24°49´	97°56´	8	8
**19. YJX**	Xima, Yingjiang	24°42´	97°44´	9	5
**20. YX**	Manwan, Yunxian	24°39´	100°19´	8	7
**21. ZY**	Shanjie, Zhenyuan	24°07´	101°14´	9	7
	**Total**			183	137

## Results

### Haplotype variation and neutrality tests

**Figure 1:**
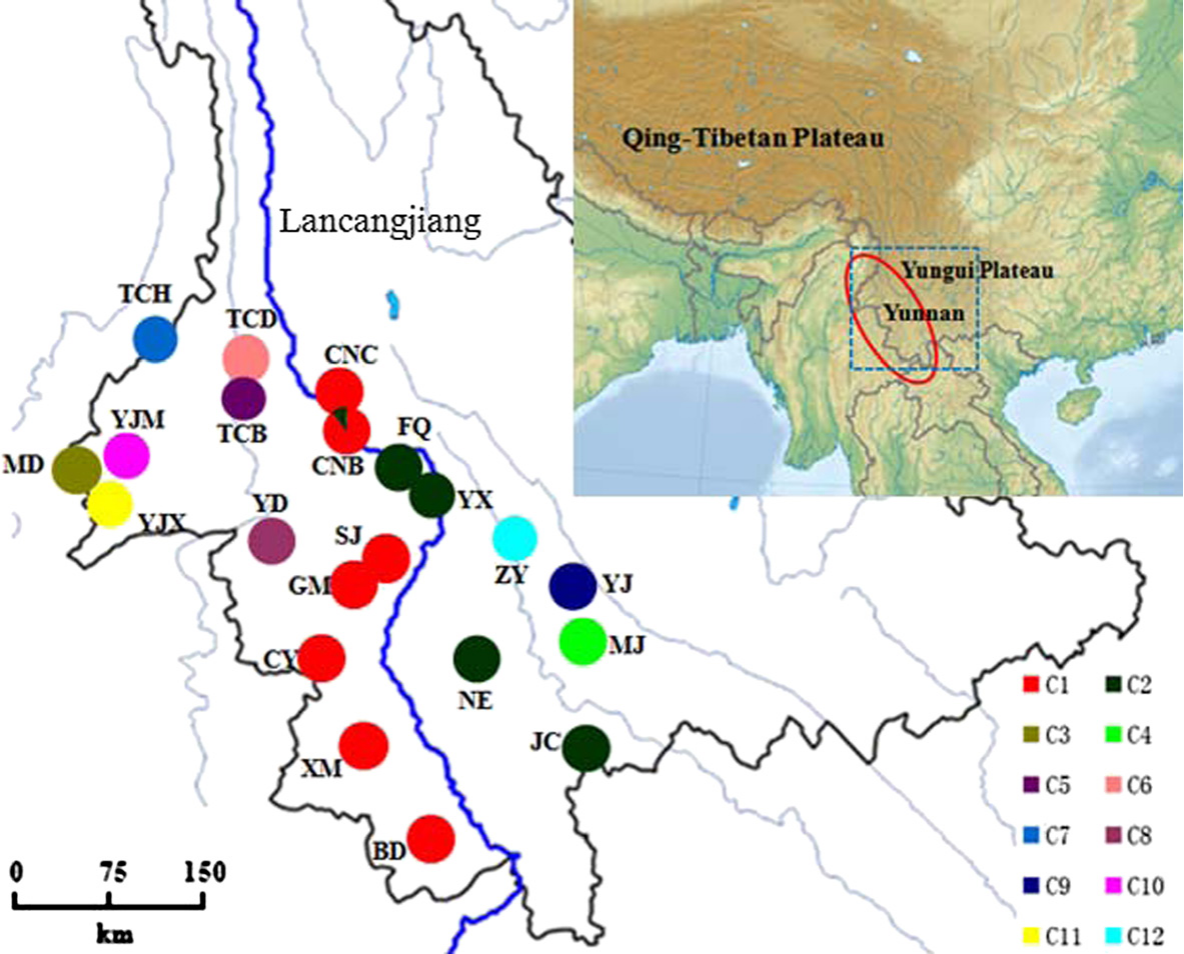
**The geographical distribution of 12 *rpl32-trnL *chlorotypes in the 21 *C. taliensis *populations. ** The Lancang River is indicated in blue, while red circle showes the range of the species.

cpDNA sequences from the *rpl32-trnL* spacer were obtained from 183 individuals of *C. taliensis* and aligned to be 858 bp in length. These sequences represented 12 haplotypes with 18 polymorphic sites, including five insertions/deletions (indels) and 13 substitutions (Table 2). Of the five detected indels, three were single nucleotide, while the other two had 5 and 9 bp, respectively (Table 2). Of the analyzed 21 populations, only one population (CNB) harbored two chlorotypes, while the other 20 were monomorphic (Table 3 and Figure 1). It is of interest to find that two chlorotypes, C1 and C2, were detected in the ten populations from the Langcang River region. Chlorotype C1 was fixed in six populations CNC, SJ, GM, CY, XM and BD, and occurred with a high frequency in the population CNB. With the exception of a low frequency occurred in the CNB population, however, Chlorotype C2 was unique to populations FQ, NE and YX from Lancang River region and one eastern population JC. Three chlorotypes (C4, C9 and C12) were present within populations located in eastern range of the species, while the remaining seven (C3, C5, C6, C7, C8, C10 and C11) were found in western populations.

**Table 2:** **Twelve haplotypes of *C. taliensis *identified in the *rpl32-trnL *sequences**

**Haplotypes**	**Polymorphic sites**																	
	**1**	**8**	**9**	**1**	**1**	**1**	**1**	**2**	**2**	**2**	**3**	**4**	**5**	**6**	**6**	**7**	**7**	**8**
	**1**	**4**	**0**	**0**	**0**	**4**	**6**	**1**	**5**	**5**	**6**	**0**	**8**	**1**	**2**	**7**	**8**	**1**
				**1**	**2**	**6**	**3**	**6**	**1**	**6**	**4**	**7**	**6**	**7**	**3**	**3**	**9**	**9**
C1	-	T	T	A	*	G	-	C	-	C	A	A	G	A	A	C	-	C
C2	T	•	G	•	*	•	-	•	-	•	•	•	•	C	•	•	-	•
C3	-	G	•	•	*	•	-	•	-	•	•	•	•	•	•	•	-	•
C4	-	•	•	•	*	•	-	•	-	•	C	•	•	•	•	•	-	•
C5	-	•	•	•	*	•	-	•	-	•	•	•	•	•	•	T	-	•
C6	-	•	•	•	*	•	-	•	-	T	•	•	•	•	•	•	-	•
C7	-	G	•	C	*	T	-	T	-	•	•	T	•	•	•	•	-	•
C8	-	•	•	•	-	•	-	•	-	•	•	•	•	•	•	•	-	•
C9	-	•	G	•	*	•	A	•	-	•	•	•	T	•	C	•	-	T
C10	-	G	•	C	*	•	-	•	T	•	•	•	•	•	•	•	-	•
C11	-	G	•	C	*	•	-	•	-	•	•	•	•	•	•	•	-	•
C12	-	•	G	•	*	•	-	•	-	•	•	•	•	•	•	•	#	•

The number at the top indicates polymorphic sites. Indels are numbered according to the first position in which they occur. Dots represent nucleotide variants identical to the first sequence; - indicates absence; *and # denote two indels (AAATA and AAATATCTA).

**Table 3:** **Chlorotype distribution and measures of haplotype diversity in *C. taliensis *populations **

**Populations**	**Haplotypes**												***S***	***n***	***h***	***π***	***K***
	**C1**	**C2**	**C3**	**C4**	**C5**	**C6**	C7	**C8**	**C9**	**C10**	**C11**	**C12**					
BD	8												0	1	0	0	0
CNB	8	1											3	2	0.22222	0.00079	0.66667
CNC	9												0	1	0	0	0
CY	10												0	1	0	0	0
FQ		9											0	1	0	0	0
GM	8												0	1	0	0	0
JC		7											0	1	0	0	0
MD			12										0	1	0	0	0
MJ				9									0	1	0	0	0
NE		7											0	1	0	0	0
SJ	7												0	1	0	0	0
TCB					10								0	1	0	0	0
TCD						10							0	1	0	0	0
TCH							8						0	1	0	0	0
XM	10												0	1	0	0	0
YD								9					0	1	0	0	0
YJ									7				0	1	0	0	0
YJM										8			0	1	0	0	0
YJX											9		0	1	0	0	0
YX		8											0	1	0	0	0
ZY												9	0	1	0	0	0
Total	60	32	12	9	10	10	8	9	7	8	9	9	18	12	0.84129	0.00314	2.6531

*n* represents the number of haplotypes, *S* represents the number of segregating sites, *h* represents haplotype diversity, *π* represents nucleotide diversity, and *K* represents average number of differences.

**Figure 2:**
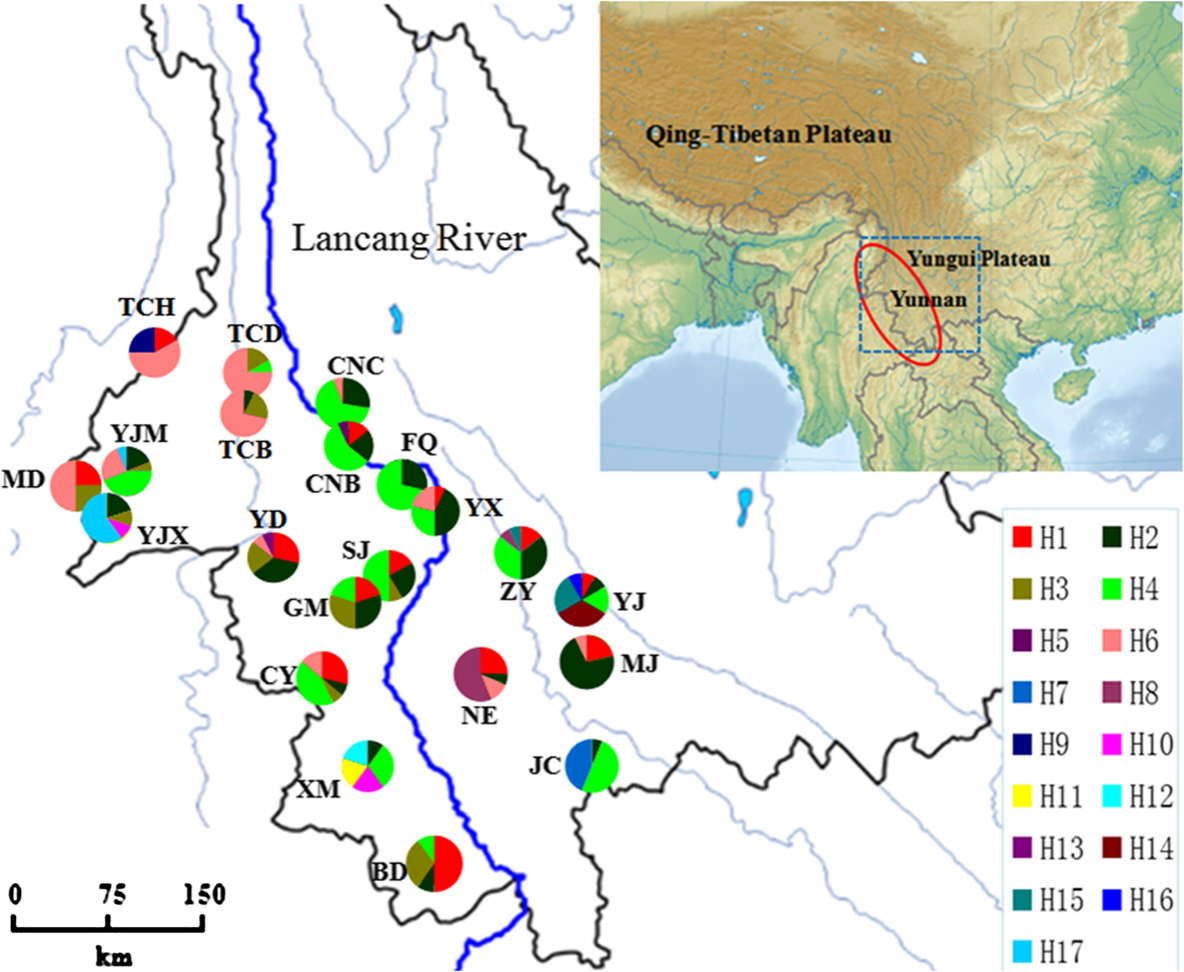
**The geographical distribution of 17 *PAL *haplotypes in the 21 *C. taliensis *populations. ** The Lancang River is indicated in blue, while red circle showes the range of the species.

The aligned nuclear *PAL* gene fragments of 627 bp were obtained from the 137 detected individuals of *C. taliensis*. With two singletons and without indels, 18 polymorphic sites were identified from 274 nrDNA sequences (Table 4). Of them, 13 polymorphic sites were synonymous, while the other five polymorphic sites were replacement changes. Among a total of 17 distinct nrDNA haplotypes found in the species, five haplotypes were common in the whole set of samples (Table 5 and Figure 2). The most frequent haplotype was H4 (observed 75 times, 27.4%), while H2 (observed 56 times, 20.4%), H6 (observed 44 times, 16.1%), and H1 (observed 34 times, 12.4%) were present in a total of 14, 18, 11 and 13 populations, respectively. In addition, Haplotype H3, which had only one mutational step from H1 and H6, was observed 20 times (7.3%) and appeared in a total of ten populations. All other haplotypes were observed 10 times or fewer (≤ 3.6%) and only present in fewer than two populations.

**Table 4:** **Seventeen haplotypes of *C. taliensis *identified in nrDNA *PAL *sequences **

**Sequence positions**																		
**Haplotypes**	**3**	**5**	**7**	**9**	**1**	**1**	**1**	**2**	**2**	**3**	**3**	**3**	**3**	**4**	**4**	**5**	**5**	**6**
	**0**	**8**	**9**	**7**	**6**	**6**	**7**	**0**	**0**	**2**	**3**	**5**	**9**	**3**	**8**	**2**	**8**	**1**
					**3**	**4**	**2**	**4**	**8**	**6**	**7**	**8**	**1**	**6**	**1**	**0**	**6**	**9**
H1	C	C	C	C	T	G	T	G	A	G	A	C	C	A	C	A	C	G
H2	•	T	•	•	C	•	•	•	G		•	•	•	G	•	•	T	•
H3	•	•	•	•	C	•	•	•	•	•	•	•	•	•	•	•	•	•
H4	•	T	•	•	C	•	•	•	•	•	•	•	•	G	•	•	T	•
H5	•	T	•	•	C	•	•	•	•	•	•	•	•	G	•	•	T	A
H6	•	•	•	•	C	•	•	•	•	•	•	T	•	•	•	•	•	•
H7	•	T	•	•	C	A	•	•	•	•	•	•	•	G	•	•	T	•
H8	•	T	•	•	C	•	•	T	•	•	•	•	•	G	•	•	T	•
H9	•	•	•	•	C	•	C	•	•	•	G	•	•	G	•	•	T	•
H10	•	T	•	•	C	•	•	•	•	•		•	•	G	T	•	T	•
H11	T	•	•	•	C	•	•	•	•	•	•	•	T	•	•	•	•	•
H12	•	T	•	•	C	•	•	•	G	•	•	•	•	G	•	C	T	•
H13	•	•	T	•	C	•	C	•	•	•	•	•	•	G	•	•	T	•
H14	•	T	•	T	C	•	•	•	•	•	•	•	•	G	•	•	T	•
H15	T	•	•	•	C	•	•	•	•	•	•	•	•	•	•	•	•	•
H16	•	•	•	•	C	•	C	•	•	•	•	•	•	G	•	•	T	•
H17	•	T	•	•	C	•	•	•	•	A	•	•		G	•	•	T	•

The number at the top indicates polymorphic sites, and dots represent nucleotide variants identical to the first sequence.

**Table 5:** ***PAL *haplotype distribution and measures of haplotype diversity in *C. taliensis *populations **

	**Haplotypes**																	***S***	***n***	***h***	***π***	***K***
**Populations**	**H1**	**H2**	**H3**	**H4**	**H5**	**H6**	**H7**	**H8**	**H9**	**H10**	**H11**	**H12**	**H13**	**H14**	**H15**	**H16**	**H17**					
BD	5	1	3	1														5	4	0.71111	0.00291	1.82222
CNB	2	3		8	1													6	4	0.64835	0.00249	1.56044
CNC		5		12		1												5	3	0.50327	0.00139	0.86928
CY	4	1	1	6		2												6	5	0.75824	0.00393	2.46154
FQ		4		10														1	2	0.43956	0.0007	0.43956
GM	2	3	3	2														5	4	0.82222	0.00397	2.48889
JC		1		8			7											2	3	0.59167	0.00104	0.65
MD	2		2			4												2	3	0.71429	0.00159	1
MJ	3	10				1												6	3	0.47253	0.00361	2.26374
NE	4	1				2		9										7	4	0.64167	0.00444	2.78333
SJ	2	3	1	6														5	4	0.71212	0.00309	1.93939
TCB			2	1		9												4	3	0.43939	0.00145	0.90909
TCD		1	3			10												5	3	0.47253	0.00161	1.01099
TCH	2					7			3									6	3	0.62121	0.00394	2.4697
XM		1		3						2	2	2						8	5	0.86667	0.00471	2.95556
YD	4	5	3			1							1					8	5	0.79121	0.00464	2.91209
YJ	1	1		2										4	3	1		8	6	0.84848	0.00462	2.89394
YJM		3	1	7		4											1	6	5	0.75	0.00355	2.225
YJX		2	1							1							6	6	4	0.64444	0.00269	1.68889
YX	1	6		4		3												6	4	0.73626	0.00375	2.35165
ZY	2	5		5				1							1			7	5	0.76923	0.0034	2.13187
Total	34	56	20	75	1	44	7	10	3	3	2	2	1	4	4	1	7	18	17	0.83639	0.00417	2.61555

*n* represents the number of haplotypes, *S* represents the number of segregating sites, *h* represents haplotype diversity, *π* represents nucleotide diversity, and *K* represents average number of differences.

To investigate whether natural selection on the *PAL* may affect the inference of population structure in *C. taliensis*, neutrality test was performed by analyzing the entire dataset. Neither Tajima*'*s *D* (*D = −*0.257, *P* > 0.1) nor Fu and Li's D* (D* = 0.52565, *P* > 0.1) rejected the null hypothesis of neutral evolution. Furthermore, we found that the minimum number of recombination events, *Rm,* was zero at *PAL*. Since *PAL* provided sufficient variation without the recombination and neutrally evolves, patterns of haplotype variation at this locus may reliably reflect the population history of *C. taliensis*.

### Population genetic structure

Levels of haplotype and nucleotide diversity were investigated at both the nuclear *PAL* and chloroplast *rpl32-trnL* across all the 21 natural populations of *C. taliensis*. On the whole, levels of total haplotype diversity and overall nucleotide diversity detected at the locus *rpl32-trnL* (*h* = 0.841; *π* = 0.00314) (Table 3) were almost as high as at the locus *PAL* (*h* = 0.836; *π* = 0.00417) (Table 5). In this study, we failed to detect sequence variation within populations except for the CNB population at the locus *rpl32-trnL* (Table 3). However, it is noticeable that genetic diversity at the locus *PAL* varied largely from one population to another, with haplotype diversity ranging from 0.439 to 0.867, and nucleotide diversity varying from 0.0007 to 0.0047 (Table 5). Among the studied populations, the XM population exhibited the most abundant genetic diversity (*h* = 0.86667; *π* = 0.00471), while the FQ population possessed the lowest levels of genetic variability (*h* = 0.43956; *π* = 0.0007).

To further investigate population genetic structure of *C. taliensis*, genetic differentiation was examined by detecting sequence variation at both the nuclear *PAL* and chloroplast *rpl32-trnL* across the 21 natural populations. Chlorotype variation revealed that population differentiation was fairly high (*G*_ST_ = 0.988), which was almost as high as the ordered alleles (*N*_ST_ = 0.989). A permutation test showed that the difference between *N*_ST_ and *G*_ST_ was not significant (U = 0.07, *P* > 0.05). Nevertheless, we found a significantly larger *N*_ST_ of 0.301 than *G*_ST_ of 0.222 detected at the locus *PAL* (U = 3.34, *P* < 0.01). Analysis of molecular variance (AMOVA) further showed that the majority of chlorotype variation (98.75%, *P* < 0.001) was found among populations, and only a small amount (1.25%) was partitioned within populations (Table 6A). A global *F*_*ST*_ value of 0.9875 also indicated fairly high differentiation among the sampled populations at the locus *rpl32-trnL*. However, AMOVA exhibited contrastingly different pattern of genetic differentiation among and within populations at the locus *PAL*. Although nucleotide diversity (77.49%) was mainly attributable to the variation within populations, significant proportion of diversity (22.51%, *P* < 0.001) was partitioned among populations (Table 6B). *F*_*ST*_ analysis (*F*_*ST*_ = 0.225, *P* < 0.001) also indicate that considerable differentiation existed significantly among the sampled populations.

**Table 6:** **AMOVA analysis of the 21 *C. taliensis *populations by using *rpl32-trnL *(A) and *PAL *(B) sequences **

**(A)**				
**Source of variation**	**d.f.**	**Sum of squares**	**Variance components**	**Percentage of variation (%)**
**Among populations**	20	75.668	0.43397	98.75*
**Within populations**	162	0.889	0.00549	1.25
**Total**	182	76.557	0.43946	
**(B)**				
**Source of variation**	**d.f.**	**Sum of squares**	**Variance components**	**Percentage of variation (%)**
**Among populations**	20	31.327	0.09512	22.51*
**Within populations**	253	82.841	0.32743	77.49*
**Total**	273	114.168	0.42255	

* *P* < 0.001.

In addition, a significant correlation was detected between genetic and geographical distances (*r* = 0.234, *P* < 0.001) at the locus *PAL*, indicative of the isolation by distance between populations. Nevertheless, there was no significant correlation between genetic and geographical distances (*r* = −0.1134, *P* = 0.966) for the locus *rpl32-trnL*.

### Nested clade phylogeographic analysis

**Figure 3:**
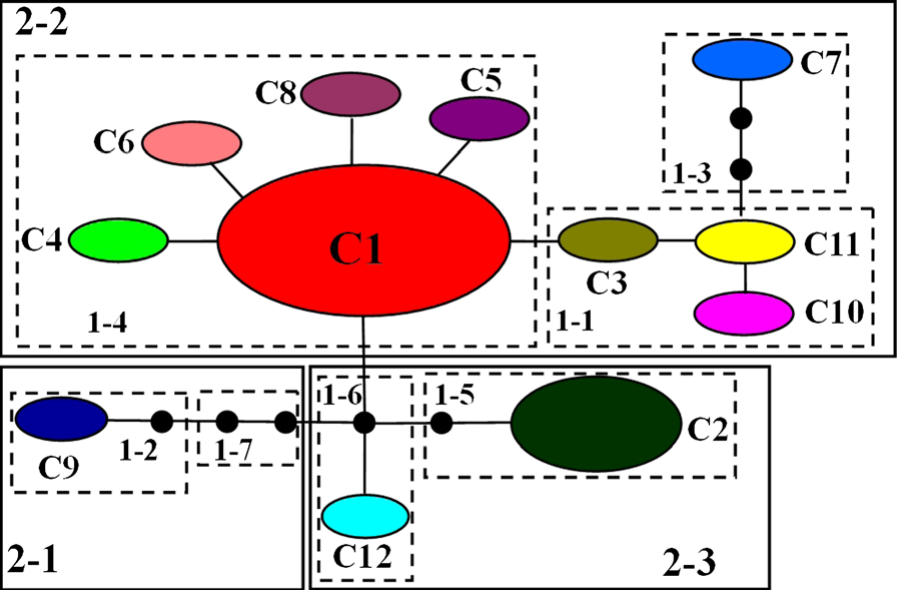
**Nested cladogram of 12 chlorotypes across the 21 *C. taliensis *populations. ** Circles in colors denote different haplotypes, and the size of each circle is proportional to that haplotype frequency across populations. Each branch between the haplotypes represents a mutational step. The dotted lines indicate independent mutation events converging on a shared haplotype. The squared loops show the nested clades or haplotypes in the network, which were resolved by nested clade phylogeographic analysis (NCPA).

A nested cladogram of chlorotypes was constructed through a TCS network by linking the haplotypes in a hierarchical manner (Figure 3). Seven one-step and three two-step clades were revealed with five clades that were significant under NCPA. In this study, we detected allopatric fragmentation in Clades 1–1 and 2–3. Clade 1–1 included chlorotypes C3, C10 and C11 sampled from the westernmost range of the species, while Clade 2–3 consisted of chlorotypes C2 and C12 distributed in eastern populations of the species. Clade 1–4 showed a restricted gene flow but with some extent of long-distance dispersal, and they distributed in the Lancang River region (Chlorotype C1), western (chlorotypes C5, C6 and C8) and eastern range (Chlorotype C4), respectively. As for Clade 2–2, we detected restricted gene flow/dispersal but with some extent of long-distance dispersal over intermediate areas not occupied by the species or past gene flow/dispersal followed by the extinction of intermediate populations. From the entire cladogram, it was inferred that they might have arisen via long-distance colonization and/or past fragmentation in (Additional file 1: Table S1).

**Figure 4:**
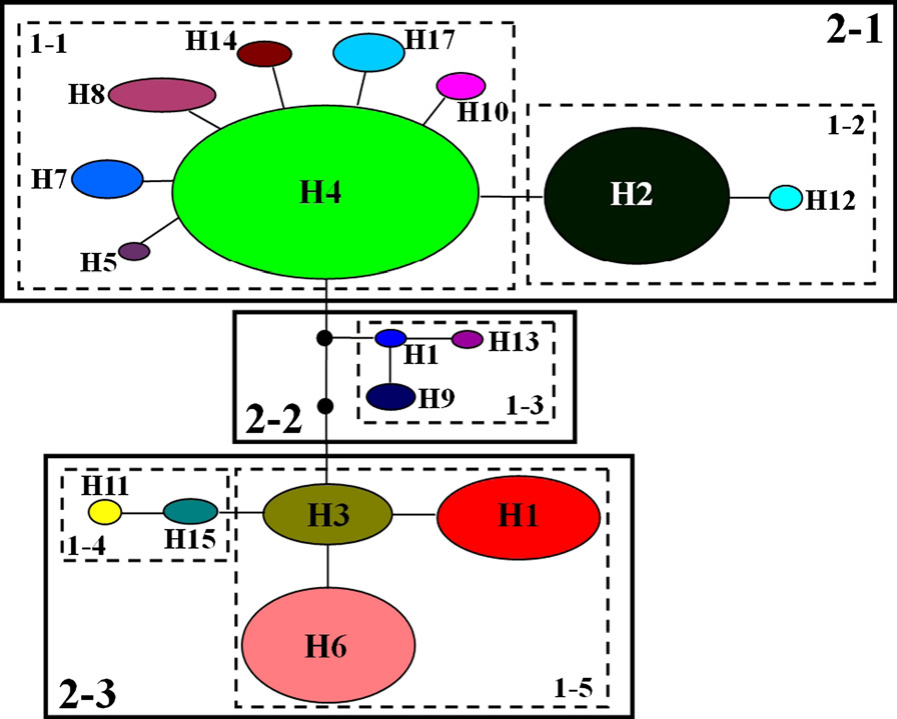
**Nested cladogram of 17 nrDNA haplotypes across the 21 *C. taliensis *populations. ** Circles in colors denote different haplotypes, and the size of each circle is proportional to that haplotype frequency across populations. Each branch between the haplotypes represents a mutational step. The dotted lines indicate independent mutation events converging on a shared haplotype. The squared loops show the nested clades or haplotypes in the network, which were resolved by nested clade phylogeographic analysis (NCPA).

The conversion of statistical parsimony network for nrDNA haplotypes into a hierarchical nested design resulted in five one-step and three two-step clades (Figure 4). In total, NCPA showed that four clades were significant. In Clades 1–1 and 1–5, we detected restricted gene flow/dispersal but with some long-distance dispersal over intermediate areas not occupied by the species, or past gene flow followed by extinction of intermediate populations. Contiguous range expansion could account for the distribution of genetic variation within the Clade 2–3. Meanwhile, restricted gene flow with isolation by distance could be inferred, which might have shaped patterns observed within the total cladogram in (Additional file 2: Table S2).

The net pairwise cpDNA divergence (dA) between three pairs of two-level clades was estimated to be 0.006245 (2–1 vs 2–2), 0.004753 (2–1 vs 2–3) and 0.001488 (2–1 vs 2–3), respectively. Assuming 1.0-3.0 × 10^-9^ s/s/y for synonymous cpDNA in seed plants [24], clade 2–1 and 2–2 might diverge approximately 1.04-3.12 MYA, the time of divergence between clade 2–1 and 2–3 could be dated to 0.79-2.38 MYA, and the divergence time between clade 2–2 and 2–3 was estimated to be 0.24-0.74 MYA.

## Discussion

### Genetic diversity and population structure

Of the 34 regions surveyed in the chloroplast genomes, *rpl32-trnL* intergenic spacer was previously proven to be suitable for the population-level phylogenetic studies [17], and thus has been successfully applied in phylogeography analyses of plants [18,19]. In comparison to mean estimate of cpDNA diversity (*h*_*T*_ = 0.67) detected by various cpDNA markers in 170 plant species [4], our study showed that *C. taliensis* possessed an abundant variation in the chloroplast *rpl32-trnL* intergenic with cpDNA diversity (*h*) of 0.841. In addition, levels of total haplotype diversity and overall nucleotide diversity within natural populations of *C. taliensis* at *rpl32-trnL* (h = 0.841; π = 0.00314) (Table 3) were higher than the three cultivated populations of *C. taliensis* (h =0.610, π = 0.00225), indicating the reduction of genetic diversity during the domestication [25]. However, nucleotide diversity within *C. taliensis* in this study appears as high as that of nine cultivated populations of *C. sinensis* var. *assamica* from Yunnan, China (h =0.728, π = 0.00469) [25].

By using *rpl32-trnL* intergenic spacer and *PAL* gene fragment as markers, we estimated genetic structure of *C. taliensis* populations across its distribution range in China. cpDNA data suggest that the differentiation among the *C. taliensis* populations was rather high (*G*_ST_ = 0.988; *N*_ST_ = 0.989), placing it among the surveyed plant species with the highest cpDNA differentiation [4]. Partitioning of genetic variability showed that, on average, merely 1.25% of cpDNA variation was distributed within *C. taliensis* populations and up to 98.75% among populations (Table 6A). In comparison with the above-described cpDNA data, it is of interest to uncover that nrDNA *PAL* data showed a contrastingly different genetic structure of *C. taliensis* populations. Genetic differentiation (*G*_ST_ = 0.222, *N*_ST_ = 0.301) shows that, on average, up to 69.9-77.8% of nrDNA variation was partitioned within *C. taliensis* populations and merely 22.2-30.1% among populations. The estimates were slightly higher than the mean value of *G*st of 0.184 for the other 77 angiosperms species [4]. In this study, we found that *N*_ST_ was significantly higher than *G*_ST_, suggesting that pairs of different nrDNA haplotypes from the same population have more similar sequence than pairs of different haplotypes from markedly different populations. AMOVA analysis further revealed that the majority of *PAL* nucleotide diversity (77.49%, *P* < 0.001) was significantly attributable to the variation within populations (Table 6B). The lack of genetic differentiation in the nuclear genes probably results from ancestral polymorphisms maintained by a larger effective population size, or high dispersal possibilities of nuclear genes [5]. As for the wild tea tree of *C. taliensis*, it is likely that smaller effective population size of organelle DNA than nuclear DNA results in strong genetic drift and high levels of population differentiation [4,5]. Because of the nature of cpDNA maternal inheritance in angiosperms, seed dispersal often plays an important role in shaping population genetic structure of maternally inherited cpDNA. *C. taliensis* usually generates heavy nut fruit with short-distance seed dispersal, and thus, rather limited abilities of seed dispersal among populations might lead to the observation of high cpDNA population subdivision*.*

### Demographic history of *C. Taliensis*

NCPA in this study indicates that restricted gene flow and effects of the past fragmentation appear to be of significance in together shaping the observed patterns of chlorotype variation in *C. taliensis*. Allopatric fragmentation was apparently detected in clades 1–1 and 2–3 of the chlorotype network. The most likely explanation is that the species has recently suffered the degradation and fragmentation of natural habitats in consequence of recent human overexploitation to subtropical forests. Our field surveys particularly found that, driven by market incentives, a number of natural populations of *C. taliensis* have been seriously destroyed and thus become endangered in small effective population sizes caused by over-picking of organic leaves from natural populations of *C. taliensis* (Gao and Liu, unpublished data). Moreover, the range of *C. taliensis* in China covers western region of Yungui Plateau, which is adjacent to the southeast of Qinghai-Tibetan Plateau with an average elevation of approximately 4,500 m above sea level, the largest and highest plateau in the world [26]. The extremely complex topography and climates were formed during the uplift of the Plateau especially in the southeastern region of Qinghai-Tibetan and Yungui plateaus. As a result, significant increase in geological and ecological diversity has largely enhanced rapid divergence and speciation in small and isolated populations [27]. In addition to the recently fragmented habitats as a result of human destruction, the estimated range of separation times of 0.24-3.12 MYA among clades in this study post-dates the most recent uplift the Tibetan Plateau around 3.4 MYA [28,29]. The species may have experienced habitat fragmentation possibly as a result of the uplift of Qinghai-Tibetan Plateau and subsequent larger-scale drainage. The past fragmentation may have resulted in the observed chlorotype structure of *C. taliensis,* although there is a lack of direct evidence to strongly support such an association of geographical patterns with the unspecified historical events. NCPA also detected the restricted gene flow in Clade 1–4 which included individuals from a total of eleven populations. It is true that the *C. taliensis* plants often produce heavy nut fruits with short-distance seed dispersal and thus the gene flow is fairly restricted among populations. The seriously fragmented habitats together with their endangered status indeed have largely accelerated the restricted gene flow among the small surviving natural populations detected in the species. However, one important characteristics of chlorotypes distribution was that Chlorotype C1 was found in the seven populations resided in the Lancang River region without exception. Such a geographical distribution of Chlorotype C1 suggests that the Lancang River might have provided northwards or southwards natural corridors for the long-distance dispersal of *C. taliensis* in China*.*

Hybridization is expected to have served as a possible driver of the observed patterns of chlorotypes. Geographic distribution of *C. taliensis* mostly covers the extensively growing range of *C. sinensis*. As previously documented in Baiying Mountains, Yunnan Province, hybrid zone which consists of a number of populations, called as “Er’Ga’Zi Tea”, was found to have formed between these two species (Chong-ren Yang, personal communications). However, the extent and effects of hybridization which might affect levels genetic variation and patterns of geographic population structure of *C. taliensis* remains largely unsettled and stays to be further studied.

It is our discovery that populations with geographical proximity did not share closely related geographical chlorotypes. For example, although TCB and TCD populations were geographically close with only 10 km distance apart, they were fixed for the distinct chlorotypes of C5 and C6, respectively. Such a pattern of cpDNA variation may come from incomplete lineage sorting of polymorphisms. As a kind of stochastic process randomly allocating ancestral polymorphisms into different populations or species, lineage sorting has been proven to be a major factor for the lack of associations between genealogical relationships of haplotypes and their geographical distributions [2]. Another possible explanation is that the past and recent habitat fragmentation of ancestral populations has led to the observed patterns of chlorotype structure in the species.

Isolation by distance can be tested through both the correlation of genetic and geographical distances and the nested clade phylogeographic analysis [30]. In this study, Mantel tests exhibited a significant correlation between genetic and geographical distances of *PAL* haplotypes*,* supporting the isolation by distance model across the study populations in *C. taliensis*. Moreover, the total cladogram of *PAL* haplotype network also showed the evidence of restricted gene flow with isolation by distance. Overall, both the correlation of genetic and geographical distances and NCPA together demonstrated that the population genetic structure of the species fitted the model of isolation by distance. Nevertheless, Mantel’s tests failed to detect significant correlations between genealogical relationships of chlorotypes and geographical distances. The discrepancy between the nrDNA *PAL* and cpDNA phylogeography of *C. taliensis* could result from different transmission mechanisms of nuclear and organelle genes, and/or their different tempos of lineage sorting through drift [5].

### Implications for the germplasm conservation

As the most popular non-alcoholic beverage throughout the world, a large number of tea germplasms have been collected and *ex situ* preserved in China, Japan, India and Kenya [31]. Unlike cultivated tea varieties, their wild relatives have cold tolerance and are resistant to common diseases infecting cultivated tea tree, and thus they constitute valuable gene resources for local and international tea tree improvement programs in the future. Although efforts have been made to preserve cultivated tea germplasms, it is vital that more attention to be paid to the conservation of their wild relatives has been largely neglected so far. Knowledge of genetic variation between and within populations of rare and endangered species is extremely useful for making appropriate management strategies directed towards their conservation [32]. Of these wild species, *C. taliensis* is one of the most important wild relatives of the cultivated tea and is subject to increasing threats as a result of the overexploitation and deforestation. The uncovered genetic profile presented here not only helps to gain important insights into genetic structure of *C. taliensis* populations, but also has critical implications for taking appropriate strategies of the conservation and germplasm management.

Comprehensive understanding of regional genetic structure of *C. taliensis* in this study is required to design an appropriate conservation scheme. In view of abundant genetic diversity resided within *C. taliensis* populations, an appropriate strategy for both germplasm sampling and developing *in situ* conservation of those populations with a higher variation on behalf of different geographical regions is needed. In order to capture the considerable genetic variation harbored among populations, *ex situ* germplasm collection should have sufficient sample size from each population. Since at least 22% genetic diversity of *PAL* nucleotide diversity distributed among populations, germplasm collections should be sampled from extensive geographic origins. The observation that the majority of *PAL* variation was distributed within populations of this species is instructive for adopting a plan of involving fewer populations but more individuals within populations. Apparently, the populations with abundant haplotype diversity, such as XM, YJ, GM, YD, ZY, CY, and NE (Table 5), may be more attractive for both *in situ* conservation and *ex situ* germplasm collections. The populations with the unique *PAL* haplotypes, such as CNB, JC, TCH, XM, YD and YJ, should be given conservation priority (Table 5). The observed chlorotype structure showed an allopatric fragmentation in *C. taliensis* such as between Clades 1–1 and 2–3. Notably, three chlorotypes (C3, C11 and C10) distributed in the westernmost range of *C. taliensis*, while other two (C2 and C12) was found in eastern populations of the species (Figure 3), further implying that both germplasm sampling and setting *in situ* conservation localities should take different geographical origins and the observed chlorotype structure into consideration.

Considering that the most remnant populations of *C. taliensis* are turning into smaller as a result of human destruction, however, it is quite possible that the process of habitat fragmentation will lead to a loss of genetic diversity by dramatically increasing mating opportunities between relatives within small populations. The *C. taliensis* populations may have suffered habitat fragmentation due to either the uplift of Tibetan Plateau or recent deforestation, and thus brought about the observed chlorotype structure in *C. taliensis.* However, NCPA suggest that restricted gene flow/seed dispersal may have resulted in smaller effective population sizes of the outcrossing plant species due to the recently fragmented habitats and long-distance colonization. Consequently, conservation and restoration genetics should concentrate on the maintenance of historically significant processes such as strong gene flow/seed dispersal as well as large effective population size in the species.

## Conclusions

Our phylogeographic study has revealed abundant genetic diversity and moderate genetic differentiation of natural populations of *C. taliensis* from the whole range of China. The data are indicative of taking effective conservation actions of these precious tea tree germplasm. However, it should be noted that samples from the entire range of the species are not represented in the present study. Therefore, a full picture of population genetic structure for the species as well as further understanding of evolutionary history and forces could be better outlined if extensive studies are completed in the seeing future. In addition, further detailed studies on reproductive biology should also help to explain the observed population structure of the wild tea tree species. Undoubtedly, such efforts will be critical for taking effective conservation of precious genetic resources of wild *C. taliensis*. Last of all, conservation consideration should be set to habitat management because human destruction of the species’ habitats has led to the species’ endangerment. Recent human destruction of wild *C. taliensis* populations driven by economic incentives plus the overexploitation and deforestation in subtropical regions have together led to the degradation and fragmentation of habitats suitable for natural populations of these species. Thus, the genetic diversity of remaining populations of the species has to be dynamically maintained in changeable environments, and long-term habitat protection is the most important to prevent genetic variation from further loss and a reduction of effective population size. Without a shred of doubt, successful conservation will largely rely on the scale of protection range of natural habitats and indeed the amount of positive participation by governments and local communities.

## Methods

### Material sampling and DNA extraction

In this study, a total of 185 individual plants from 21 wild populations of *C. taliensis* were sampled, representing almost the entire range of the species in China (Table 1; Figures 1 and 2). For each population, young, healthy leaves were collected from seven to thirty individual trees, depending on population size. To ensure adequate population coverage, random samples were taken from trees at an interval of about 3–100 m across the whole studied population. Voucher specimens were collected and deposited in the Herbarium of Kunming Institute of Botany, Chinese Academy of Sciences (KUN). Leaves were individually harvested in the field and silica-dried for subsequent DNA extractions. Genomic DNA was then isolated from liquid-nitrogen ground leaf tissues according to a CTAB method described by Doyle and Doyle [33].

#### PCR amplification and DNA sequencing

The extracted DNA was dissolved in 100 μL TE buffer and then used as the PCR templates. In this study, primer pairs used by Shaw et al. [17] were adopted to amplify the *rpl32-trnL* noncoding spacer of cpDNA (*TRNL*: 5' -CTG CTT CCT AAG AGC AGC GT -3', *RPL32*: 5'-CAG TTC CAA AA A AAC GTA CTT C-3'). In addition, a pair of primers (*PAL* F: 5'-TGC CAC AAT CAG CCA CAA G-3', *PAL* R: TGG TTG GTT ACA GGA TTG GC) was designed based on cDNA sequence from *C. sinensis* due to the unavailability of the entire genomic structure of *PAL* in *C. taliensis*.

DNA amplifications were carried out in a T1 thermocycler (Biometra), programmed for an initial 4 mins at 97 °C; followed by 35 cycles of 50 s at 95 °C, 50 s at 52 °C (*rpl32-trnL*) or 55 °C (*PAL*), 1 min at 72 °C; and an additional extension for 10 mins at 72 °C. Reactions were performed in 50 μL reaction volumes containing 50 mM KCl, 10 mM Tris–HCl (pH 8.3), 1.5 mM MgCl_2_, 200 μM of each dNTP, 0.1 μM of each primer, 30–50 ng of template DNA, and 1U *Taq* polymerase (TaKaRa). Amplification products were run on 0.8% agarose gels and subsequently purified with UNIQ-10 kit (Shanghai Bioengineering). PCR products were sequenced by ABI 3730 at Beijing Genomics Institute (BGI) –Shenzhen.

Although *PAL* was reported to be a single-copy nuclear gene in *C. sinensis*, it is possible that lineage-specific duplication occurred in *C. taliensis*. To exclude the possibility of paralogous loci in *C. taliensis*, we selected and cloned 15 heterozygous PCR products using pGEM T-easy vectors. For every selected heterozygous PCR product from a single individual, as expected for the amplification of a single locus from a diploid organism, no more than two different alleles were detected. Therefore, it was confirmed that all these haplotypes detected in our data set correspond to the same *PAL* locus. All sequences reported in this study were deposited in the GenBank database under accession numbers JX161616-JX161644.

### Data analyses

Because some of the SNPs used in this study were discovered within close proximity to one another, they could not be treated as independent markers. For each set of linked SNP loci, we employed a Bayesian statistical method implemented in Phase version 2.1.1 [34,35] to resolve the gametic phase of PAL sequences with multiple heterozygous single nucleotide polymorphisms (SNPs). This program uses allele frequencies and frequencies of known SNP haplotypes in each population to infer the probabilities for each possible haplotype from a group of linked SNPs. A total of five independent runs of 100 iterations each were performed with other parameters as default. The goodness-of-fit values were very similar among different runs, indicating that their run lengths were sufficient in the present study. For each newly ‘phased’ locus, we selected the two haplotypes for each sample that had the highest probability as assessed by PHASE. These haplotypes were then used as multi-allelic genotypes for further analysis. Only those alleles and genotypes resolved with > 95% posterior probabilities were remained for subsequent analyses. Sequences were proofed and aligned by using CLUSTAL _X [36] as implemented in BioEdit [37]. Indels in the cpDNAs were treated as substitutions by following Caicedo and Schaal [2].

Global and population nucleotide diversity (*π*) [38], haplotype diversity (*h*), average number of nucleotide differences between the whole sequences (*K*), and the number of polymorphic sites (*S*) were calculated using DNASP 4.10 [39]. Tajima's *D*[40] and Fu & Li's D*[41] neutrality tests were applied to determine whether a locus evolves in a neutral manner. The minimum number of recombination events (*R*_M_) was assessed using the algorithm of Hudson and Kaplan [42] in the DNASP 4.10 program.

Nested clade phylogeographic analysis (NCPA) was performed by following the approach [43] in the program ANeCA [44]. Significantly parsimonious connections were then constructed by using the program TCS [45], with a 95% parsimony connection limit. On basis of the resulting network, nested clades were further defined following the rules of Templeton et al. [46] and Templeton & Sing [47]. In the study, the program GEODIS [48] was used to test whether there is geographic associations of clades as well as nested clades or not under the null hypothesis, with a 95% confidence level and with 10,000 permutations. If significant values were detected, the inference key of Templeton [49] was used to explain their likely population processes and/or historical events within these clades.

The approximate divergence times between clades defined by nested clade phylogeographic analysis were estimated following Yuan et al. [50], using T = d_A_/2 μ,where T is the divergence time and μ is the rate of nucleotide substitution [51]. The net pairwise divergence per base pair (d_A_) was calculated using MEGA4 [52] under the Kimura two-parameter model [53]. In this study, considering that a substitution rate had not yet been estimated for the cpDNA genome of *Camellia*, 1.0-3.0 × 10^-9^ substitutions per site per year for synonymous cpDNA sites in seed plants [24], were taken as a rough evolutionary rate for *rpl32-trnL* intergenic spacers to date their divergence times.

An analysis of molecular variance (AMOVA) [54] was carried out with Arlequin 3.1 [55] to determine the partitioning of variation within and between populations. Two measures of population differentiation *G*_ST_ and *N*_ST_ were compared by using U-statistic implemented by the program HAPLONST [56]. *G*_ST_ values were estimated by haplotype frequencies, while *N*_ST_ was obtained by considering similarities between haplotypes (i.e. the number of mutations between haplotypes). An *N*_ST_ which is significantly larger than a *G*_ST_, indicates the presence of a phylogeographical structure with closely related haplotypes being detected more frequently in the same area than remotely correlated ones.

The Mantel test implemented in the program Arlequin 3.1 [55] was applied to examine the correlation between the natural logarithm of the geographical distance and Slatkin's measure *MM* = (1/*F*_ST_ − 1)/2], a measure of the extent of gene flow under an island model at equilibrium [57]. Statistical significance was also tested with 10, 000 permutation tests by using the program Arlequin 3.1.

## Abbreviations

AMOVA: Analysis of molecular variance; NCPA: Nested clade phylogeographic analysis; PAL: Phenylalanine ammonia-lyase; SNP: Single nucleotide polymorphisms.

## Authors’ contributions

YL collected population samples, generated experimental data, performed the whole data analyses, and drafted the earlier versions of the manuscript. SXY and PZJ involved the field sample collection. LZG designed the study, partially analyzed the data, wrote and revised the manuscript. All of the authors have read and approved the final manuscript.

## Supplementary Material

Additional file 1 **Table S1. ** Chain of inference from the nested clade analysis of the chlorotype data in *C. taliensis* using Templeton’s (2004) inference key. Click here for file

Additional file 2 **Table S2.** Chain of inference from the nested clade analysis of the *PAL* haplotype data in *C. taliensis* using Templeton’s (2004) inference key. Click here for file

## References

[B1] Hajjar R Hodgkin T The use of wild relatives in crop improvement: A survey of developments over the last 20 years Euphytica 2007 156 1 13 10.1007/s10681-007-9363-0

[B2] Caicedo AL Schaal BA Population structure and phylogeography ofSolanum pimpinellifolium inferred from a nuclear gene Mol Ecol 2004 13 1871 1882 10.1111/j.1365-294X.2004.02191.x 15189210

[B3] Maxted N Scholten M Codd R Ford-Lloyd B Creation and use of a national inventory of crop wild relatives Biol Conserv 2007 140 142 159 10.1016/j.biocon.2007.08.006

[B4] Petit RJ Duminil J Fineschi S Hampe A Salvini D Vendramin GG Comparative organization of chloroplast, mitochondrial and nuclear diversity in plant populations Mol Ecol 2005 14 689 701 15723661 10.1111/j.1365-294X.2004.02410.x

[B5] Schaal BA Hayworth DA Olsen KM Rauscher JT Smith WA Phylogeographic studies in plants: problems and prospects Mol Ecol 1998 7 465 474 10.1046/j.1365-294x.1998.00318.x

[B6] Avise JC Phylogeography: the history and formation of species 2000 Harvard University Press, Cambridge, London

[B7] Besnard G Khadari B Baradat P Bervillé A Olea europaea(Oleaceae) phylogeography based on chloroplast DNA polymorphism Theor Appl Genet 2002 104 1353 1361 10.1007/s00122-001-0832-x 12582591

[B8] de Alencar Figueiredo LF Calatayud C Dupuits C Billot C Rami JF Brunel D Perrier X Courtois B Deu M Glaszmann JC Phylogeographic evidence of crop neodiversity in sorghum Genetics 2008 179 997 1008 10.1534/genetics.108.087312 18558653 PMC2429892

[B9] Londo JP Chiang YC Hung KH Chiang TY Schaal BA Phylogeography of Asian wild rice,Oryza rufipogon, reveals multiple independent domestications of cultivated rice,Oryza sativa Proc Nat Acad Sci 2006 103 9578 9583 10.1073/pnas.0603152103 16766658 PMC1480449

[B10] Muller MH Poncet C Prosperi JM Santoni S Ronfort J Domestication history in theMedicago sativaspecies complex: inferences from nuclear sequence polymorphism Mol Ecol 2006 15 1589 1602 10.1111/j.1365-294X.2006.02851.x 16629813

[B11] Olsen KM Schaal BA Evidence on the origin of cassava: phylogeography ofManihot esculenta Proc Nat Acad Sci 1999 96 5586 5591 10.1073/pnas.96.10.5586 10318928 PMC21904

[B12] Xu X Ke W Yu X Wen J Ge S A preliminary study on population genetic structure and phylogeography of the wild and cultivatedZizania latifolia(Poaceae) based onAdh1asequences Theor Appl Genet 2008 116 835 843 10.1007/s00122-008-0717-3 18283426

[B13] Ming TL Monograph of the genusCamellia 2000 Yunnan Science and Technology Press, Kunming

[B14] Burban C Petit RJ Phylogeography of maritime pine inferred with organelle markers having contrasted inheritance Mol Ecol 2003 12 1487 1495 10.1046/j.1365-294X.2003.01817.x 12755877

[B15] Wang HW Ge S Phylogeography of the endangeredCathaya argyrophylla(Pinaceae) inferred from sequence variation of mitochondrial and nuclear DNA Mol Ecol 2006 15 4109 4122 10.1111/j.1365-294X.2006.03086.x 17054506

[B16] Zarza E Reynoso VH Emerson BC Diversification in the northern neotropics: mitochondrial and nuclear DNA phylogeography of the iguanaCtenosaura pectinataand related species Mol Ecol 2008 17 3259 3275 10.1111/j.1365-294X.2008.03826.x 18564087

[B17] Shaw J Lickey EB Schilling EE Small RL Comparison of whole chloroplast genome sequences to choose noncoding regions for phylogenetic studies in angiosperms: the tortoise and the hare III Amer J Bot 2007 94 275 288 10.3732/ajb.94.3.275 21636401

[B18] Falchi A Paolini J Desjobert JM Melis A Costa J Varesi L Phylogeography of Cistus creticus L. on Corsica and Sardinia inferred by the TRNL-F and rpl32-trnL sequences of cpDNA Mol Phylogenet Evol 2009 52 538 543 10.1016/j.ympev.2009.04.002 19364536

[B19] Sosa V Ruiz-Sanchez E Rodriguez-Gomez FC Hidden phylogeographic complexity in the Sierra Madre Oriental: the case of the Mexican tulip poppyHunnemannia fumariifolia(Papaveraceae) J Biogeogr 2009 36 18 27 10.1111/j.1365-2699.2008.01957.x

[B20] Matsumoto S Takeuchi A Hayatsu M Kondo S Molecular cloning of phenylalanine ammonia-lyase cDNA and classification of varieties and cultivars of tea plants (Camellia sinensis) using the teaPALcDNA probe Theor Appl Genet 1994 89 671 675 10.1007/BF00223703 24178009

[B21] Matsumoto S Kiriiwa Y Takeda Y Differentiation of Japanese green tea cultivars as revealed by RFLP analysis of phenylalanine ammonia-lyase DNA Theor Appl Genet 2002 104 998 1002 10.1007/s00122-001-0806-z 12582605

[B22] Kaundun S Matsumoto S Development of CAPS markers based on three key genes of the phenylpropanoid pathway in Tea, Camellia sinensis (L.) O. Kuntze, and differentiation between assamica and sinensis varieties Theor Appl Genet 2003 106 375 383 12589537 10.1007/s00122-002-0999-9

[B23] Kaundun SS Matsumoto S PCR-based amplicon length polymorphisms ALPs at microsatellite loci and indels from non-coding DNA regions of cloned genes as a means of authenticating commercial Japanese green teas J Sci Food Agr 2004 84 895 902 10.1002/jsfa.1665

[B24] Wolfe KH Li WH Sharp PM Rates of nucleotide substitution vary greatly among plant mitochondrial, chloroplast, and nuclear DNAs Proc Nat Acad Sci 1987 84 9054 9058 10.1073/pnas.84.24.9054 3480529 PMC299690

[B25] Liu Y Yang SX Gao LZ A comparative study on the chloroplastRPL32-TRNLnucleotide variation within and genetic differentiation among ancient tea plantations ofCamellia sinensisvar. assamicaandC. taliensisfrom Yunnan, China Acta Bot Yunnanica 2010 32 427 434

[B26] Zheng D The system of physico-geographical regions of the Qinghai-Xizang (Tibet) Plateau Sci China Ser D 1996 39 410 441

[B27] Liu JQ Wang YJ Wang AL Hideaki O Abbott RJ Radiation and diversification within the Ligularia–Cremanthodium–Parasenecio complex (Asteraceae) triggered by uplift of the Qinghai-Tibetan Plateau Mol Phylogenet Evol 2006 38 31 49 10.1016/j.ympev.2005.09.010 16290033

[B28] Sun H Zheng D Formation, evolution and development of Qinghai-Xizang (Tibetan) plateau 1998 Guangdong Science and Technology Press, Guangzhou

[B29] Cheng J Liu X Gao Z Tang D Yue J Effect of the Tibetan Plateau uplifting on the geological environment of the Yunnan Plateau Geoscience 2001 15 290 296

[B30] Cuenca A Escalante AE Piñero D Long-distance colonization, isolation by distance, and historical demography in a relictual Mexican pinyon pine (Pinus nelsoniiShaw) as revealed by paternally inherited genetic markers(cpSSRs) Mol Ecol 2003 12 2087 2097 10.1046/j.1365-294X.2003.01890.x 12859631

[B31] Chen L Gao Q Chen D Xu C The use of RAPD markers for detecting genetic diversity, relationship and molecular identification of Chinese elite tea genetic resources [Camellia sinensis(L.) O. Kuntze] preserved in a tea germplasm repository Biodivers Conserv 2005 14 1433 1444 10.1007/s10531-004-9787-y

[B32] Milligan BG Leebens-Mack J Strand AE Conservation genetics: beyond the maintenance of marker diversity Mol Ecol Notes 1994 3 423 435

[B33] Doyle JJ Doyle JL A rapid DNA isolation procedure for small quantities of fresh leaf tissue Phytochem Bull 1987 19 11 15

[B34] Stephens M Smith NJ Donnelly P A new statistical method for haplotype reconstruction from population data Am J Hum Genet 2001 68 978 989 10.1086/319501 11254454 PMC1275651

[B35] Stephens M Donnelly P A comparison of bayesian methods for haplotype reconstruction from population genotype data Am J Hum Genet 2003 73 1162 1169 10.1086/379378 14574645 PMC1180495

[B36] Thompson JD Gibson TJ Plewniak F Jeanmougin F Higgins DG The CLUSTAL_X windows interface: flexible strategies for multiple sequence alignment aided by quality analysis tools Nucl Acid Res 1997 25 4876 4882 10.1093/nar/25.24.4876 PMC147148 9396791

[B37] Hall TA BioEdit: a user-friendly biological sequence alignment editor and analysis program for Windows 95/98/NT Nucleic Acids Symp Ser 1999 41 95 98

[B38] Nei M Li WH Mathematical model for studying genetic variation in terms of restriction endonucleases Proc Nat Acad Sci 1979 10 5269 5273 291943 10.1073/pnas.76.10.5269 PMC413122

[B39] Rozas J Sanchez-DelBarrio JC Messeguer X Rozas R DnaSP, DNA polymorphism analyses by the coalescent and other methods Bioinformatics 2003 19 2496 2497 10.1093/bioinformatics/btg359 14668244

[B40] Tajima F Statistical method for testing the neutral mutation hypothesis by DNA polymorphism Genetics 1989 123 585 595 2513255 10.1093/genetics/123.3.585 PMC1203831

[B41] Fu YX Li WH Statistical tests of neutrality of mutations Genetics 1993 133 693 709 8454210 10.1093/genetics/133.3.693 PMC1205353

[B42] Hudson RR Kaplan NL Statistical properties of the number of recombination events in the history of a sample of DNA sequences Genetics 1985 111 147 164 4029609 10.1093/genetics/111.1.147 PMC1202594

[B43] Templeton AR Maxwell T Posada D Stengard JH Boerwinkle E Sing CF Tree scanning: a method for using haplotype trees in phenotype/genotype association studies Genetics 2005 169 441 453 15371364 10.1534/genetics.104.030080 PMC1448891

[B44] Panchal M Beaumont MA Sunnucks P The automation and evaluation of nested clade phylogeographic analysis Evolution 2007 61 1466 1480 10.1111/j.1558-5646.2007.00124.x 17542853

[B45] Clement M Posada D Crandall KA TCS: a computer program to estimate gene genealogies Mol Ecol 2000 9 1657 1659 10.1046/j.1365-294x.2000.01020.x 11050560

[B46] Templeton AR Boerwinkle E Sing CF A cladistic analysis of phenotypic associations with haplotypes inferred from restriction endonuclease mapping. I. Basic theory and an analysis of alcohol dehydrogenase activity in Drosophila Genetics 1987 117 343 351 2822535 10.1093/genetics/117.2.343 PMC1203209

[B47] Templeton AR Sing CF A cladistic analysis of phenotypic associations with haplotypes inferred from restriction endonuclease mapping. IV. Nested analyses with cladogram uncertainty and recombination Genetics 1993 134 659 669 8100789 10.1093/genetics/134.2.659 PMC1205505

[B48] Posada D Crandall KA Templeton AR GeoDis: a program for the cladistic nested analysis of the geographical distribution of genetic haplotypes Mol Ecol 2000 9 487 488 10.1046/j.1365-294x.2000.00887.x 10736051

[B49] Templeton AR Statistical phylogeography: methods of evaluating and minimizing inference errors Mol Ecol 2004 13 789 809 10.1046/j.1365-294X.2003.02041.x 15012756

[B50] Yuan QJ Zhang ZY Peng H Ge S Chloroplast phylogeography ofDipentodon(Dipentodontaceae) in southwest China and northern Vietnam Mol Ecol 2008 17 1054 1065 10.1111/j.1365-294X.2007.03628.x 18208489

[B51] Nei M Kumar S Molecular evolution and phylogenetics 2000 Oxford University Press, Oxford

[B52] Tamura K Dudley J Nei M Kumar S MEGA4: molecular evolutionary genetics analysis (MEGA) software version 4.0 Mol Biol Evol 2007 24 1596 1599 10.1093/molbev/msm092 17488738

[B53] Kimura M A simple method for estimating evolutionary rates of base substitutions through comparative studies of nucleotide sequences J Mol Evol 1980 16 111 120 10.1007/BF01731581 7463489

[B54] Excoffier L Smouse PE Quattro JM Analysis of molecular variance inferred from metric distances among DNA haplotypes: application to human mitochondrial DNA restriction data Genetics 1992 131 479 491 1644282 10.1093/genetics/131.2.479 PMC1205020

[B55] Excoffier L Laval G Schneider S Arlequin (version 3.0): an integrated software package for population genetics data analysis Evol. Bioinform. Online 2005 1 47 50 19325852 PMC2658868

[B56] Pons O Petit RJ Measuring and testing genetic differentiation with ordered versus unordered alleles Genetics 1996 144 1237 1245 8913764 10.1093/genetics/144.3.1237 PMC1207615

[B57] Slatkin M Isolation by distance in equilibrium and non-equilibrium populations Evolution 1993 47 264 279 10.2307/2410134 28568097

