# Supplementary material for: Phylogeography of Camellia taliensis (Theaceae) inferred from chloroplast and nuclear DNA: insights into evolutionary history and conservation
Source: BMC Evol Biol. 2012 Jun 21;12:92. doi: 10.1186/1471-2148-12-92 (PMC3495649; doi:10.1186/1471-2148-12-92)
Supplement: Additional file 1 — Table S1. Chain of inference from the nested clade analysis of the chlorotype data in C. taliensis using Templeton’s (2004) inference key. [file 1471-2148-12-92-S1.doc]

**Sup Table 1**

**Chain of inference from the nested clade analysis of the chlorotype data in *C. taliensis* using Templeton’s (2004) inference key***

| **Clade** | **Permutational**  **chi-squared statistic** | **Probability** | **Clade key** | **Inferences** |
| --- | --- | --- | --- | --- |
| Clade 1-1 | 58.0000 | 0.0000 | 1-19 NO | Allopatric fragmentation |
| Clade 1-4 | 392.000 | 0.0000 | 1-19-20-2-3-5-6-7 YES | Restricted gene flow/dispersal but with some long-distance dispersal |
| Clade 2-2 | 270.000 | 0.0000 | 1-19-20-2-3-5-6*-7-8 YES | Restricted gene flow/dispersal but with some long-distance dispersal over intermediate areas not occupied by the species; or past gene flow followed by extinction of intermediate populations |
| Clade 2-3 | 41.0000 | 0.0000 | 1-19 NO | Allopatric fragmentation |
| Total Cladogram | 360.8276 | 0.0000 | 1-2-11-12-13-14 NO | Long-distance colonisation and/or past fragmentation (not necessarily mutually exclusive) |

***** Data presented here only for the clades with significant association between haplotype and geography (*P <* 0.05)
