# Supplementary material for: Phylogeography of Camellia taliensis (Theaceae) inferred from chloroplast and nuclear DNA: insights into evolutionary history and conservation
Source: BMC Evol Biol. 2012 Jun 21;12:92. doi: 10.1186/1471-2148-12-92 (PMC3495649; doi:10.1186/1471-2148-12-92)
Supplement: Additional file 2 — Table S2. Chain of inference from the nested clade analysis of the PAL haplotype data in C. taliensis using Templeton’s (2004) inference key. [file 1471-2148-12-92-S2.doc]

**Additional_file_2.pdf Table S2**

**Chain of inference from the nested clade analysis of the *PAL* haplotype data in *C. taliensis* using Templeton’s (2004) inference key***

| **Clade** | **Permutational**  **chi-squared statistic** | **Probability** | **Clade key** | **Inferences** |
| --- | --- | --- | --- | --- |
| Clade 1-1 | 332.7446 | 0.0000 | 1-2-3-5-6-7-8 YES | Restricted gene flow/dispersal but with some long-distance dispersal over intermediate areas not occupied by the species; or past gene flow followed by extinction of intermediate populations |
| Clade 1-5 | 65.2911 | 0.0004 | 1-2-3-5-6-7-8 YES | Restricted gene flow/dispersal but with some long-distance dispersal over intermediate areas not occupied by the species; or past gene flow followed by extinction of intermediate populations |
| Clade 2-3 | 77.9410 | 0.0000 | 1-2-11-12 NO | Contiguous range expansion |
| Total Cladogram | 153.4814 | 0.0000 | 1-2-3-4 NO | Restricted gene flow with isolation by distance |

***** Data presented here only for the clades with significant association between haplotype and geography (*P <* 0.05)
